# The Microcellular Structure of Injection Molded Thick-Walled Parts as Observed by In-Line Monitoring

**DOI:** 10.3390/ma13235464

**Published:** 2020-11-30

**Authors:** Dariusz Sykutera, Piotr Czyżewski, Piotr Szewczykowski

**Affiliations:** Department of Manufacturing Techniques, Faculty of Mechanical Engineering, UTP University of Science and Technology, Kaliskiego 7, 85-796 Bydgoszcz, Poland; piotr.czyzewski@utp.edu.pl (P.C.); piotr.szewczykowski@utp.edu.pl (P.S.)

**Keywords:** microcellular injection molding, MuCell^®^ technology, pressure sensor, Priamus system, in-line monitoring, special mold cavity, apparent viscosity, reinforced PA66 GF30 composite, thick-walled parts, micro CT analysis

## Abstract

The aim of the study was to detect the influence of nitrogen pressure on the rheological properties and structure of PA66 GF30 thick-walled parts, produced by means of microcellular injection molding (MIM), using the MuCell^®^ technology. The process was monitored in-line with pressure and temperature sensors assembled in the original injection mold. The measured data was subsequently used to evaluate rheological properties inside an 8.4 mm depth mold cavity. The analysis of the microcellular structure was related to the monitored in-line pressure and temperature changes during the injection process cycle. A four-times reduction of the maximum filling pressure in the mold cavity for MIM was found. At the same time, the holding pressure was taken over by expanding cells. The gradient effect of the cells distribution and the fiber arrangement in the flow direction were observed. A slight influence of nitrogen pressure on the cells size was found. Cells with a diameter lower than 20 µm dominate in the analyzed cases. An effect of reduction of the average cells size in the function of distance to the gate was observed. The creation of structure gradient and changes of cells dimensions were evaluated by SEM images and confirmed with the micro CT analysis.

## 1. Introduction

In recent years, the development of thermoplastic polymer injection molding technologies allowed the production of high quality products, particularly those of engineering polymers. The required quality is obtained by controlling and monitoring the manufacturing process using the Industry 4.0 system tools [1,2]. These systems can be used to analyze geometric features of a molded piece, surface condition, structure, processing, and utilizing properties.

One of the observed trends in design is minimizing the mass of products (lightweight elements), while keeping or improving some mechanical properties at the same level. Microcellular injection molding (MIM) technologies support the processing of polymeric materials towards obtaining foamed materials. Llewelyn et al. presented in their review article the significant development in different aspects of microcellular injection molding [3]. The obtained foamed parts have less weight, improved impact strength, maintained toughness, and insulating properties [3,4,5,6].

Sun et al. obtained a significant ductility growth for the microcellular structure of PP/LDPE and PLA/PHBV blends at a ratio of 75/25 and 70/30, respectively [7]. It was found that for fiber-reinforced polymers, shrinkage anisotropy, sink marks, warpage, residual stresses, and deformations were minimalized [3]. MIM processes are realized with a lower injection pressure (less clamping force needed) and the holding phase switched off, which significantly reduce the cycle time [7,8,9]. In order to minimize possible defects, especially at the surface of the molded part, the MIM technology is combined with other types of injection molding processes [3]. An example is the research conducted by Szostak et al., whose subject was the impact of the parameters of MuCell^®^ and InduMold technologies on the selected processing and mechanical properties, structure and surface quality of molded parts, and their comparison [10]. The quality improvement of the molded parts was obtained by Guo et al. in the combined processes of In-Mold Decoration and MIM [11]. Bledzki et al. combined MIM and gas counter pressure (GCP) methods to significantly reduce the molded parts surface roughness [12]. The results analysis of the research, involving assessment creating of gas pores in the structure of polymers by means of MIM, shows that they mostly concern the relationship between the process parameters, structure, and mechanical properties. Castner et al. identified a significant and non-linear impact of the degree of foaming on the mechanical properties of microcellular polypropylene parts [13]. Chang et al. showed that a faster cooling rate of polystyrene in the mold cavity causes an increase in cell density and a reduction in their pore size by over 100% [14].

Based on the papers review from the last 5 years, it can be seen that the polypropylene and its composites with EPDM, talc, or short fibers are the most popular material used for MIM. Usually, the properties and structure of foam molded parts with a wall thickness of not more than 4 mm are analyzed. An analysis of the data in Table 1 indicates that PA66 is a material of great interest for industrial application, but still rarely examined for the MIM process. Volpe et al. investigated the key process parameters of MuCell^®^ such as injection temperature, gas injection pressure, as well as morphology and mechanical properties of PA66 GF30 foamed parts (2–4 mm thick) [15]. It was found that the amount of gas and gas injection pressures increase the density reduction of PA66 GF30 parts. Moreover, the higher the gas pressure and the thinner the part wall, the smaller pores can be obtained. The core of foam parts is dominated by pores ranging in size from 10 to 50 micrometers. Authors showed that, the bigger the wall thickness of moldings, the larger the cells have grown. Therefore, it can be seen that studies of melts rheological behaviors with the dissolved supercritical nitrogen fluid are published in a low number of papers [9,16,17]. In fact, laboratory methods do not take into account the phenomena that occur during the plasticizing of polymer granulates and filling the mold cavity with a melt in a processing tool [18,19]. In this case, a measurement method is required which best reflects the characteristics of the rheological properties of the molten polymer under conditions most similar to the real processing of thermoplastics [20,21].

The solution used for measuring viscosity by Fernandez et al. was previously used in laboratory extruders [26,27]. This solution consisted of placing pressure and temperature sensors inside a special rheological nozzle, which was the last zone of the plasticizing unit of the injection molding machine. Similar methods were applied by other researchers to measure the viscosity of polymer melts [17,28,29,30], where a different number of sensors and different runner geometries were applied. A popular method of determining the ability of polymers to flow under the actual conditions was to measure the length of the solid stream of a molded piece, which was obtained in the injection phase into a spiral-shaped mold cavity. The advantage of this method is to determine the correlation between the material flowability and assumed process parameters. This method is popular and frequently modified due to its simplified nature. Equipping the forming cavity with pressure sensors is one example of the modification of this method [31,32]. Another example is the measurement proposed by Hopmann et al., which concerned the real-time viscosity measurement based on the records of pressure sensors placed in the manifold of the hot-runner system [33].

In recent years, the studies on the actual relationship between process parameters and the apparent viscosity of the melted polymers, has led to applying the construction of injection tools with various mold cavity geometries in rheological studies [9]. Friesenbichler’s team in their research [34] showed that the apparent viscosity of a polypropylene melt determined with a special rheological form differs by 10% as compared to the viscosity determined by using a capillary rheometer. In other studies, the Chen’s team indicated the need to prepare dedicated rheological models based on the true injection molding process in the case of simulating thin-walled products by the high speeds injection molding process [35]. Similar studies were carried out by Sykutera et al. [18]. Gordon et al. applied an injection mold of their own construction for rheological studies [36]. A set of various pressure and temperature sensors has been arranged in the runner channel and the cavity along the entire flow length. Rheological models created by using numerical simulations based on capillary rheometer data are less accurate than rheological models obtained during the injection process under real conditions.

The actual conditions recorded by pressure and temperature sensors in the forming cavity can be used not only to determine the apparent viscosity, but also to precisely monitor and record the complete cycle of the injection process [37,38,39]. It can be used in the manufacturing of polymer parts with high quality requirements. Recording of signals by pressure and temperature sensors in the forming cavity can be correlated with changes in selected processing parameters set on the machine to optimize the injection molding process [40,41]. As a result, it is possible to automatically eliminate defective molded pieces by a comparative analysis of signals from the mold cavity and reference data for a good product [42,43,44]. Although there is a partial convergence of signals generated in the processing machine and the injection mold, the data obtained from the forming cavity are more informative and may be of greater importance [45]. Placing the sensors system for monitoring conditions in a real mold cavity gives the possibility of complete verification of analyses using the finite elements method (FEM) tools for assumed processing conditions [46].

Our extensive literature review shows that articles concerning the MuCell^®^ technology application to modify PA66 GF30 are rather rarely published. Another research by the authors concerned the foamed small size samples, which ranged in 2–4 mm thickness. The advantage of our studies is the investigation of two more thick samples (exceeding 8 mm thickness). Temperature and pressure sensors are hardly ever applied inside the mold cavity for microcellular injection molding research using the MuCell^®^ technology. Therefore, determining rheological parameters and pressure curves based on the output data directly from the mold cavity for the MIM process is very rare. In our research, we used as many as four sensors, so the process was monitored more precisely.

The aim of the study was to determine the influence of MIM technology on rheological properties of the PA66 GF30 melt determined by pressure and temperature sensors located in the mold cavity. In the study, a system to monitor pressure and temperature in a mold cavity during the filling and cooling phases of the injection molding process was applied. The relationship between nitrogen pressure, melt viscosity, and the microcellular structure obtained in the MIM process was investigated. The aim was to obtain an expected microcellular structure for thick-walled molded parts filled with a large amount of short fibers.

## 2. Material and Methods

**Figure 1:**
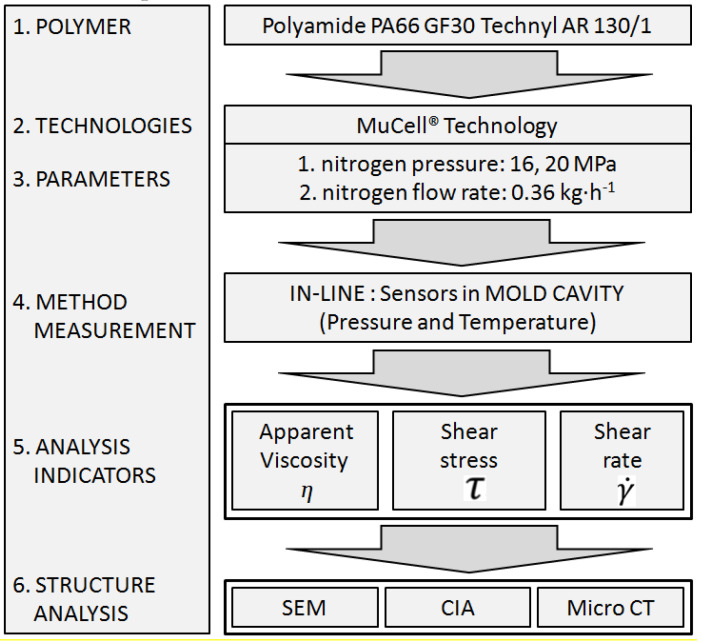
Block scheme of the experimental system with input-output data.

The experimental system with input-output data is presented in the form of a block scheme in Figure 1. The pressure and temperature measured inside the mold cavity, apparent viscosity, shear stress, and shear rate were output data.

### 2.1. Materials

Polyamide PA66 GF30 Technyl AR 130/1 (Rhodia, France) containing 30 wt% of short glass fiber (GF) was used in our investigations. The density of the polymer was 1370 kg∙m^−3^, the melting point temperature was 263 °C, and water absorption was 0.8 wt% (at 23 °C, 24 h). The polymer was dried in a vacuum chamber at 80 °C for 4 h, before use.

### 2.2. Sample and Testing Specimen Preparation

The foam parts were produced by microcellular injection molding using the MuCell^®^ technology (Trexel, MA, USA). Molded parts were fabricated by means of the Engel 500 (Engel, Austria) injection molding machine (courtesy of the GRAFORM company, Bydgoszcz, Poland). The processing conditions used during sample production in the standard injection molding and MuCell^®^ technology are summarized in Table 2. The parameters of the nitrogen in the supercritical state used in the MIM technology are depicted in Table 3. The method of injection molding and the value of nitrogen pressure were the basis of marking the process and samples with letters A, B, C (Table 2). In our investigations, the N_2_ gas was dosed for 7 s, for both pressure values. For each of the process settings, 20 measurements (injection molding cycles) were performed. After changing the process parameters the first batch of samples was not taken for further consideration.

**Figure 2:**
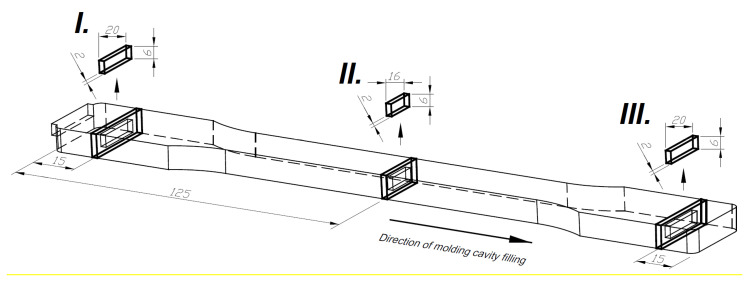
Sampling areas for structural testing (all dimensions are given in millimeter).

The influence of the MIM process parameters on the microcellular structure of PA66 GF30 parts based on SEM and micro CT images was evaluated. The samples for structural tests were taken from the sections of the foam parts, as shown in Figure 2. According to our research program, samples were taken from the following areas: Close by the gate (I), midpoint of the measurement area (II), and the end of the polymer flow path (III). The selection of the sampling place takes into account the change in the cross-section of the mold cavity.

Parts of c.a. 50 mm in length were cut out from the region of interest of the injection molded pieces. Two notches were cut on the opposite sides of the sample. Each sample was placed for one minute in liquid nitrogen and mechanically broken. Thin pieces with a revealed fracture surface were cut out and covered with platinum by ion beam stuttering.

### 2.3. Mold Settings

**Figure 3:**
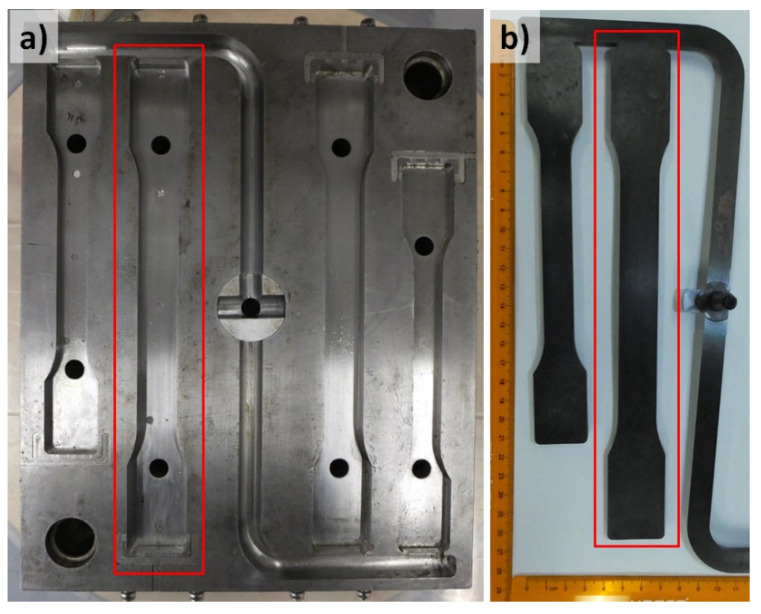
View of the mold (**a**) which was used to produce the test samples (**b**).

In the presented study, a four-cavity injection mold was used to produce thick-walled molded pieces (Figure 3a). The MIM process was monitored in the mold cavity marked with a red frame in Figure 3a. The molded parts reveal twice the dimensions compared to the specimens according to PN-EN ISO 527-2 type 1B (Figure 3b).

**Figure 4:**
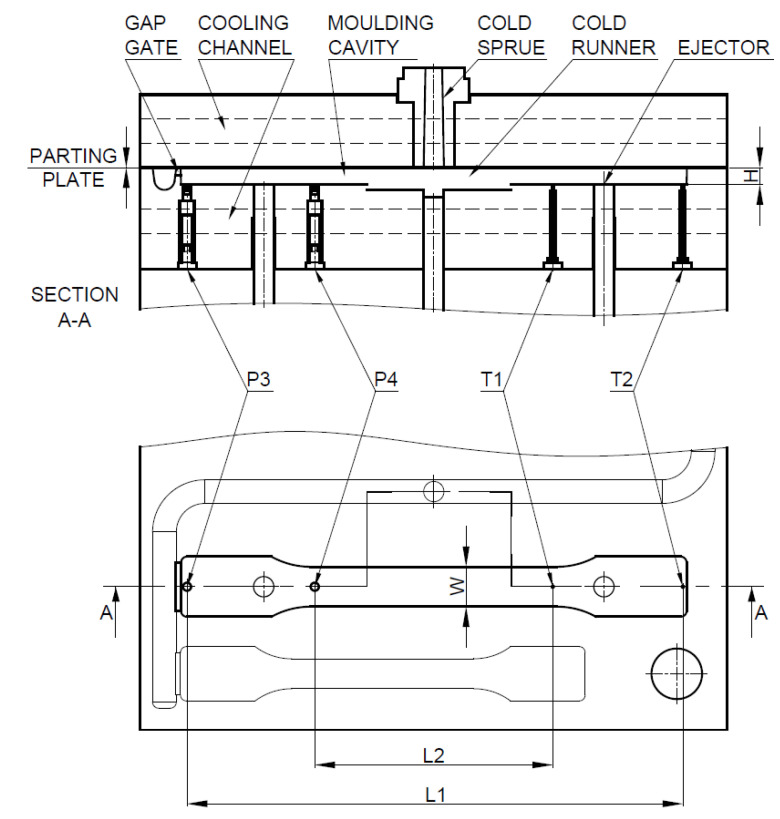
The dimensions of the cavity and the positions of pressure and temperature sensors in the larger cavity.

The mold body was made of 1.1730 steel with a hardness of 190 HB. The forming plates were made of 1.2312 steel with a hardness of 30 ± 2 HRC and had dimensions of 290 mm × 235 mm. The runner was 183 mm long and had a section in the shape of a parabola with a width of 11 mm. Gap gates of 24 mm wide and 4.2 mm deep were applied in the cavity. Cooling channels were made in both molding plates symmetrically, relatively to the mold parting plane. The cooling channels were made parallel to flow direction of the melt in the mold cavities. Two pressure sensors type 6002B (sensitive 4.93 pC/bar) and two temperature sensors type 4008B (thermocouple type N) were used. These elements of the measuring system were assembled in the bigger mold cavity (Figure 4). Two temperature and two pressure sensors applied inside the mold cavity were marked as T1, T2 and P3, P4, respectively (Figure 4). Temperature sensors were mainly used for the detection of a flow position. The pressure sensor P3 was located directly behind the gates. The temperature sensor T2 was located at the end of the melt flow path. The distance between these sensors was L1 = 245 mm (Figure 4). A second pair of sensors (P4 and T1) was located in the measuring section of the specimen (the smallest width 20 mm) in a distance of L2 = 118 mm. Locating the second pair of sensors in the mold cavity guarantees a stable flow of the polymer melt through the constant cross-section. Other dimensions of the mold cavity are marked as follows: W = 20 mm (width of the mold cavity in the P4 and T1 sensors assembly), H = 8.4 mm (depth of the mold cavity with assembled sensors). Measurements were recorded during the filling and cooling phases. The voltage signals were recorded and processed by the eDAQ^TM^ 8102 (Priamus, Switzerland) transducer (converter).

### 2.4. Experimental Method

#### 2.4.1. Rheology

The evaluation of selected rheological properties of the polymer melt, by means of an algorithm based on the in-line registration of pressure and temperature directly in the mold cavity, was realized in our studies.

**Figure 5:**
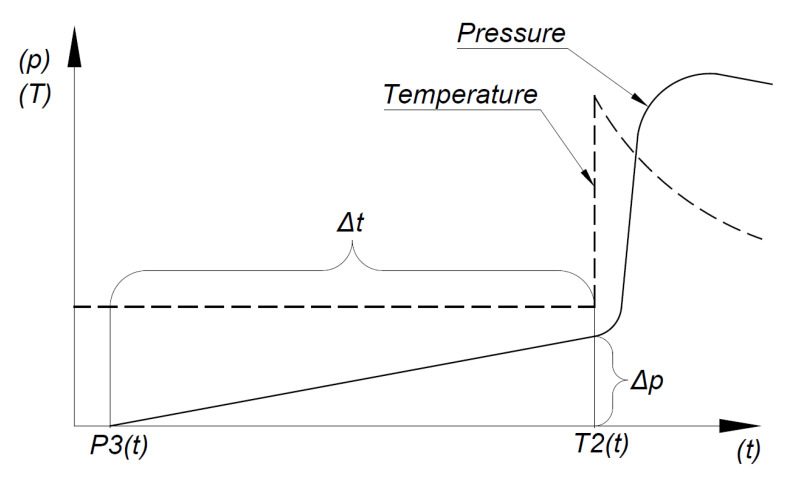
Pressure changes with time during the flow of the polymeric melt in the cavity on the L1 distance between sensors P3 and T2.

The principle of the apparent viscosity determination in the injection mold by the measurement of pressure and temperature in the mold cavity is presented in Figure 5. Data are recorded using pressure and temperature sensors placed on the measuring length *L_i_*. The measurement system begins to register the pressure and temperature each time the control system of the injection machine emits a signal corresponding to the closing stage of the mold. A proper measurement of Δ*p* corresponds to changes of pressure between the first pressure increase and the moment of receiving a signal from the temperature sensor (Δ*T*). The measured values of Δ*T* and Δ*p* are subsequently used to determine the values of shear rate ($\dot{\gamma }$), shear stress (*τ*), and apparent viscosity (*η*).

The shear stress was evaluated according to the following formula:

(1) $$\tau_{i}=\frac{\Delta p\cdot H}{2\cdot L_{i}}$$

where Δ*p* is the pressure increase between signals from pressure and temperature sensors, *H* is the height of the mold cavity (Figure 4), and *L_i_* is the distance between proper pressure and temperature sensors in the mold cavity (Figure 4).

The shear rate is described by the following equation:

(2) $$\dot{\gamma_{i}}=\frac{6\cdot Q_{i}}{W\cdot H^{2}}$$

where *Q_i_* is the flow rate of the polymer melt in the mold cavity and *W* is the width of the cavity mold.

Expressing the flow rate of the polymer melt in the mold cavity by the following equation:

(3) $$Q_{i}=\frac{L_{i}\cdot W\cdot H}{\Delta t}$$

where Δ*t* is the time of flow of molten polymer between the sensors.

The formula for shear rate takes the form of:

(4) $$\dot{\gamma_{i}}=\frac{6\cdot L_{i}}{\Delta t\cdot H}$$

According to this, the apparent viscosity, which is the ratio of shear stress to shear rate can be expressed by the following equation:

(5) $$\eta_{i}=\frac{\tau }{\dot{\gamma }}=\frac{\Delta p\cdot \Delta t\cdot H^{2}}{12\cdot {L_{i}}^{2}}$$

#### 2.4.2. Structure

Pictures of the fracture surface were taken using the JEOL 5600 scanning electron microscope at a 1 kV acceleration voltage. The approximate pore size was calculated using the ImageJ ver. 1.52a program.

Micro CT images were obtained by means of a Bruker SkyScan 1272 X-ray micro computed tomography (Kontich, Belgium) with an image pixel size of 4.5 µm, by a source voltage of 60 kV. Projections were reconstructed by the NRecon program, while the analysis was done using the CT Analyzer program. The micro CT analysis procedure for the foamed PA6 GF30 was presented by Szewczykowski and Skarżyński [47].

## 3. Results and Discussion

The addition of inert nitrogen in a supercritical state changes the rheological properties of the molted PA66 GF30 during its flow in the mold cavity. Changes of the shear stress and shear rate (Table 3) recorded by the Priamus system are the key points in the discussion on the apparent viscosity of the PA66 GF30 melt containing nitrogen. The addition of supercritical nitrogen caused a significant increase in the shear rate of a polymer melt. Nearly a double increase of the shear rate was observed for B and C samples at length L1 and L2. At the same time, the values of the shear rate did not change significantly.

This can be explained by the reduction of flow resistance of the melt containing nitrogen cells, as well as by the expansion of the material in the end phase of its flow. The measurement of rheological properties at length L2 (less flow volume) also indicates a significant increase in the shear rate of the melt containing the inert gas, while the shear stress remains at a level similar to the solid PA66 GF30 melt. Only in this case, sample B shows a decrease in the shear stress at length L1 (Table 4). The consequence of the recorded changes is a noticeable decrease in the apparent viscosity of a melted polymer after being added (as presented in Table 4).

It was also found that the lowest apparent viscosity value AV1 was obtained for sample B (Table 5). The changes observed in the apparent viscosity AV2 indicate a similar tendency, but the differences in the viscosity values between the solid (A) and foamed (B and C) samples are much more noticeable. The measurement carried out in the measuring part of the specimen (L2) showed that the flow resistance for sample A is over 30% greater than for other melts with dissolved gas (samples B and C, Table 5).

**Figure 6:**
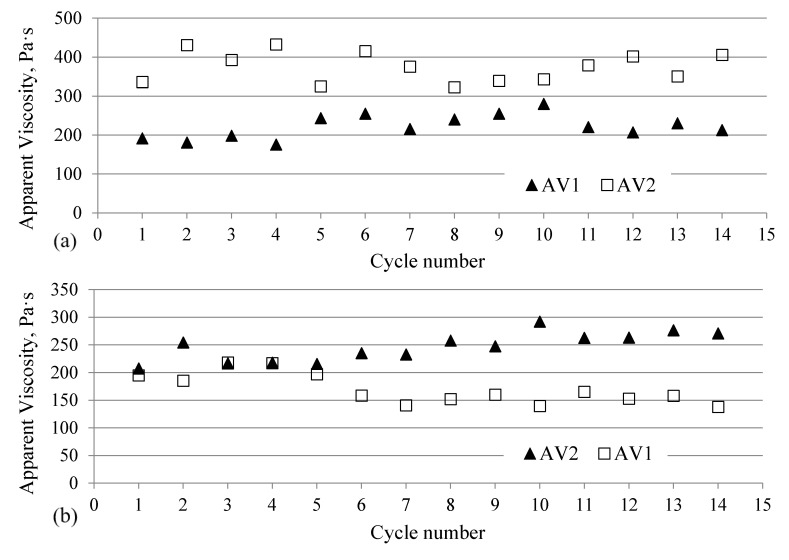

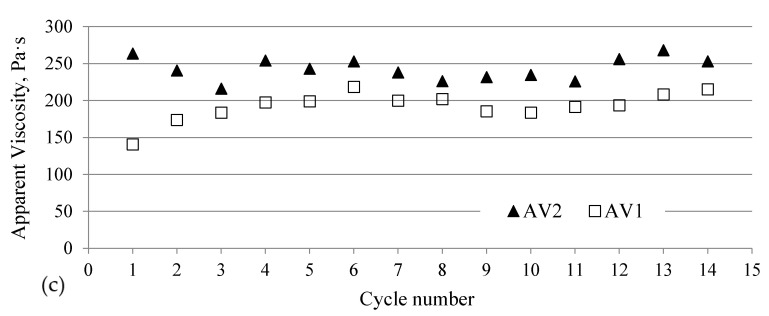
Apparent viscosity changes measured in the injection mold cavity: (**a**) For the PA66 GF30 melt—sample A, (**b**) for the PA66 GF30 melt with nitrogen—sample B, (**c**) for the PA66 GF30 melt with nitrogen—sample C.

The analysis of the standard deviation value and coefficient of variation (%) clearly indicates that the addition of inert gas to the polyamide melt containing fibers increases the repeatability of the flow parameters of the material in the injection mold cavity. This is confirmed by the graphical analysis of apparent viscosity changes in subsequent cycles of the injection process. Filling the injection mold cavity with the PA66 GF30 melt in the standard process takes place with relatively considerable changes in the apparent viscosity, thus with less repeatability of the filling of the cavity in subsequent cycles (Figure 6a). The addition of supercritical nitrogen in the MuCell^®^ technology makes this process more repeatable. This observation can be confirmed by the graph presenting the changes in apparent viscosity for samples B and C (Figure 6b,c), as well as by the analysis of the data contained in Table 5. Assuming that all other processing parameters are maintained at a constant level, this means that the molded pieces with gas pores solidify under repeatable conditions. This is confirmed by an organoleptic observation of the obtained samples. Despite the setting of a very short holding time (0.3 s), the foamed parts made in the MuCell^®^ technology well mapped the filling up of the mold cavity. All samples had sharp edges in the whole volume. It is crucial that despite significant thicknesses (over 8 mm), no warpage (shrinkage anisotropy) or surface sink marks were identified. Therefore, this confirms the previous information about the possibility of reducing the holding phase in the cycle with the help of the MuCell^®^ technology, even for molded parts with such a large thickness.

Detailed analyses of individual pressure and temperature curves in the injection mold cavity confirm the results presented above and extend the inference on the course of melt flow in the analyzed cases of the mold cavity. Figure 6a presents the pressure and temperature changes during the standard IM of a non-foamed PA66 GF30. A shape of this curve was confirmed by the literature. In principle, pressure curves recorded by sensors P3 and P4 coincide during the filling and holding phases. The maximum values of this parameter are around 24 MPa. It can also be seen that the material in the measuring point T1 cools down more slowly than in the thermocouple measuring area T2.

**Figure 7:**
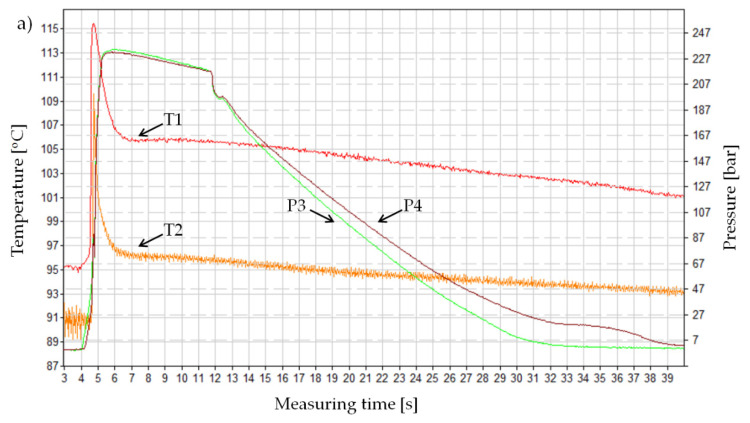

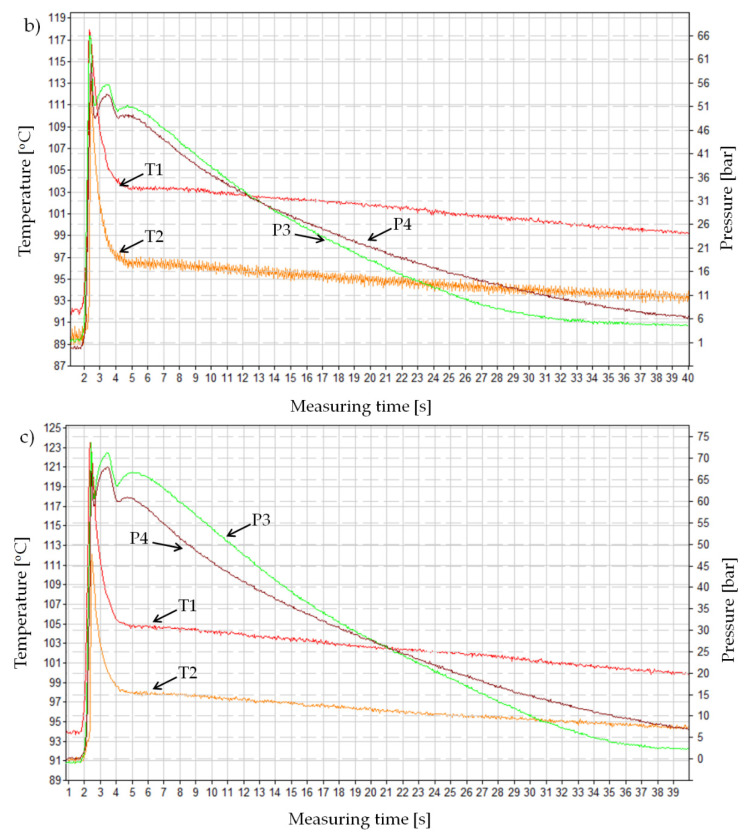
The pressure course recorded in the mold cavity during the completed cycle for: (**a**) Non-foamed process, (**b**) foamed process using the 16 MPa nitrogen pressure, (**c**) foamed process using the 20 MPa nitrogen pressure.

After adding a supercritical nitrogen in the MuCell^®^ technology, the curves change to a considerable extent (Figure 7b,c). The most significant of these changes is an approximately fourfold pressure drop in the mold cavity, regardless of the variables adopted and described in the methodological part (Table 6). It can also be seen that the pressure P4 (measured in the part of the sample with a minimized volume) is lower than in point P3 (close to the gate) and its decrease is above 1 MPa. The result obtained for sample C, both values of this parameter, are higher and the difference between them is less than 1 MPa.

**Figure 8:**
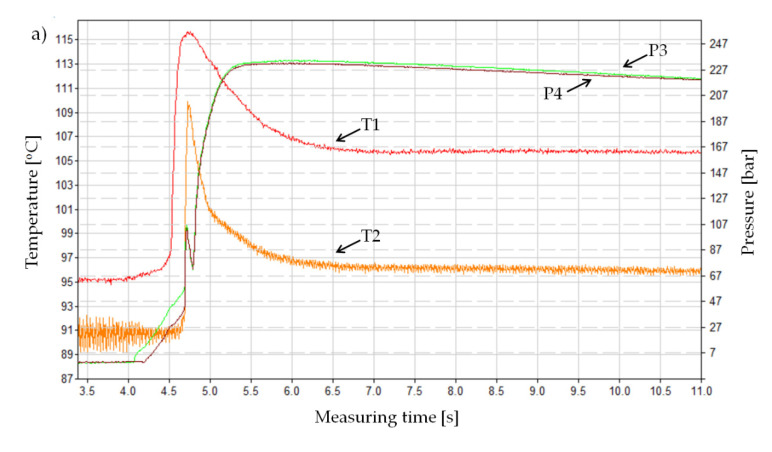

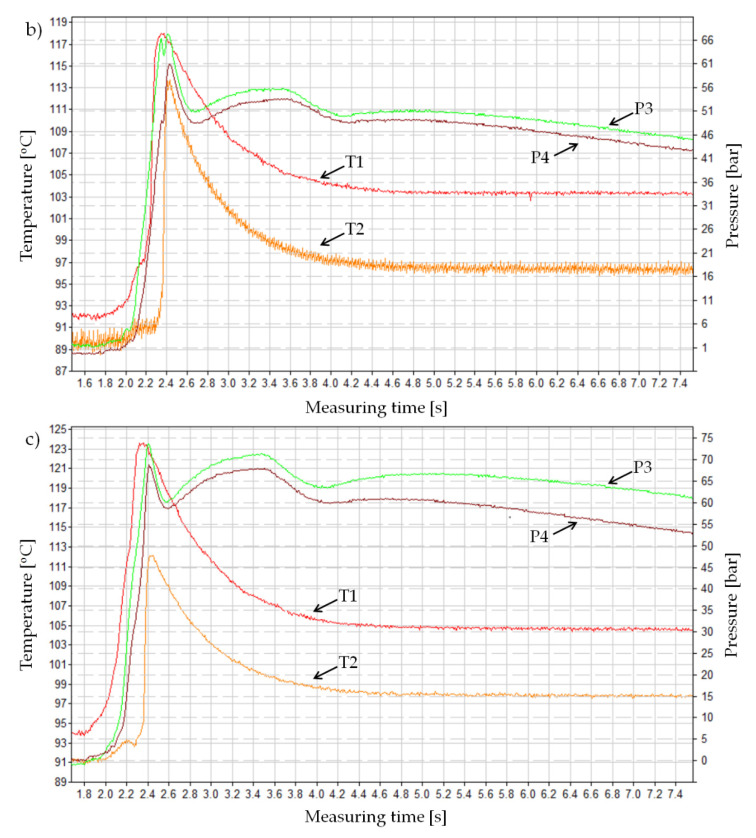
The pressure curse recorded during the filling phase and at the beginning of packing (**a**) and cooling (**b**,**c**) phases for the standard injection molding (IM) (**a**) and for the foamed process using the 16 MPa nitrogen pressure (**b**), for the foamed process using the 20 MPa nitrogen pressure (**c**).

The curves of pressure and temperature changes in the mold cavity during the cellular injection of the PA66 GF30 are different from the curves registered in the standard injection process, characteristic for semi-crystalline materials. Another difference concerns the shape of the pressure curves for all variants of the injection in the MuCell^®^ technology. An example of these changes representing the entire set is shown in Figure 7b,c and Figure 8b,c.

For samples B and C, the pressure inside the closed mold does not drop rapidly to the atmospheric pressure, despite the fact that during all tests the holding pressure lasted only for 0.3 s. This indicates the occurrence of an additional inner pressure from the core of the sample and the further growth of cells. The higher the nitrogen dosing pressure, the higher the pressure in the mold during the cooling phase. For variant C, the value of this parameter is very close to the maximum in the filling phase. This tendency can be noticed for both P3 and P4 sensors, while the value of pressure P3 is slightly higher. It is logical due to its location close to the gate. Despite the fact that pressure in the cavity is not kept constant for a long time, the thick-walled molded parts B and C were characterized by sink mark-free surfaces, appropriate dimensions, and no significant shrinkage anisotropy. For these thick-walled foamed parts deformation was not identified.

Though the absolute values of temperature obtained are not put under analysis, their nature and the repeatability of changes have a significant impact on the determination of apparent viscosity, shear stresses, and shear rates of the PA66 GF30 melt. The diagrams of the temperature change presented in Figure 7 and Figure 8 at the location of the T1 and T2 sensors confirm the different courses depending on the processing conditions. In all cases, the T1 sensor, fixed at the end of the narrowest part of the mold cavity, was the first to react to the flow of the polymer melt. Recorded by the T2 sensor, the temperature increase indicated the cavity filling of 99% by the polymer melt. The temperature maxima visible in the figures precede the peak pressure values in the mold cavity, which indicates a well realized material compression in the injection phase. A summary list of temperature changes recorded with thermocouples T1 and T2 is shown below. When the MuCell^®^ technology is used, temperature indications are highest at the T1 sensor location, which results from the fact that the melt temperature drops as the mold cavity is filled (Table 7).

After the material is visibly compressed in the measuring part of the specimen (length L2), it expands as a result of the increase in a flow cross-section (a width increase from 20 to 30 mm). This moment of the flow is clearly visible, since the temperature T1 suddenly increases. The second maximum pressure is related to a complete filling of the cavity and the subsequent compression of the melt, accompanied by an increase in the temperature of the measured thermocouple T2. This is confirmed by the known fact that during the flow of the polymer melt filled with glass fibers, additional heat is generated to the melt due to the glass fibers friction against the metal walls of the mold.

**Figure 9:**
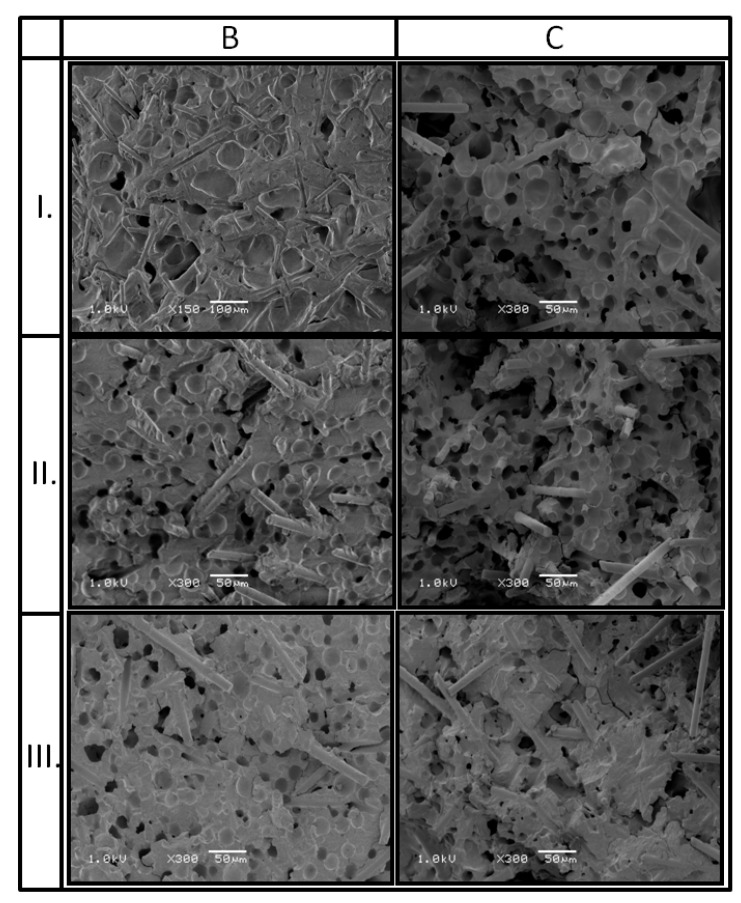
Microcellular structure of PA66 GF30 foamed samples taken in section I, II, and III for test specimens B and C.

Numerous fine cells were found in the structure of the molded parts for both samples B and C. Their average size depends on the distance of the analyzed section from the gate, which was observed on the SEM images for samples with such significant thickness compared to the literature data, this result can be considered interesting (Table 8 and Figure 9). In the case of sample B, close by the gate, the largest cells with a diameter of 60 μm were noted, while in the grip part of the sample (far from the gate), the cells diameter does not exceed 20 μm. Visible black, deep holes of 10 µm in diameter c.a. in the SEM images are traces of glass fibers (Figure 9). In the measuring area of B and C specimens (section II), similar values of the average cells size were recorded at the level of about 20 μm. In section III, located far from the gate, the pore dimensions and their shape are very similar to section II and do not exceed 20 μm as well.

Those good results of the pore size range can be explained by minimization of the fountain effect during the melt flow [48,49,50], as well as by low shear stresses during the flow and a viscosity decrease. It was caused by a lower flow rate gradient compared to the thin-walled molded part. A rapid solidification of the surface layer was obtained. All the effects of splitting the pores into smaller ones inside the sample were the result of the interaction of pores with glass fibers and the individual melt layers velocity gradient during filling of the mold cavity. The highest intensity of splitting the pores into smaller ones takes place in the region where the polymer melt enters the measuring part of the test specimen. This is caused by compressing the polymer melt, and therefore, increasing shear stresses. As a result, the effect of gas pressure on the pore size was registered only in sections I, which is consistent with the other authors’ research. The pore dimensions are similar in sections II and III.

**Figure 10:**
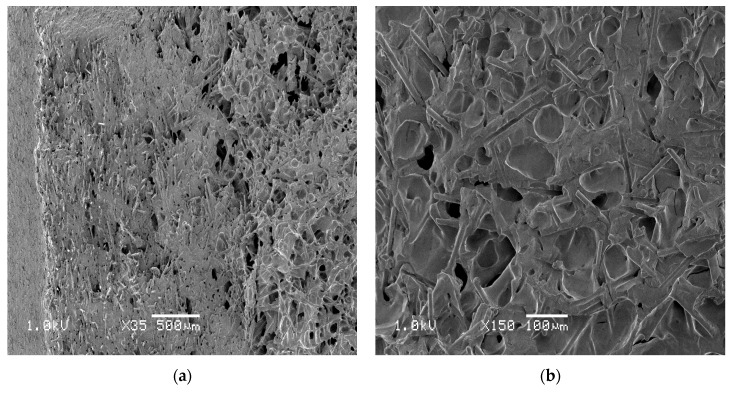
Additional morphological effects noticed during the analysis of SEM images for the B sample: (**a**) Structure gradient and fiber position, mag. ×35, (**b**) pore clusters near the end of the fibers, mag. ×150.

The analysis of SEM images confirms previous reports [3] about other fiber reinforced thermoplastics materials such as polypropylene and poly(butylene terephthalate), according to which glass fibers are elements of the structure, around which cells accumulate. The higher the percentage of glass fibers in a composite, the easier it is to obtain the microcellular structure consisting of fine pores. A gradient structure was observed for both porous samples, in terms of pores distribution (its intensity) and in terms of glass fibers direction and distribution (Figure 10). It was estimated, based on SEM images, that the outer layer of 1.4 mm does not consist of any pores, the intermediate layer was around 2.0 mm thick, while a core, with the most chaotic fibers orientation was only 1.8 mm thick. In the two first regions, glass fibers are oriented in a flow direction. For both kinds of investigated samples, glass fibers are more chaotically oriented in a core part. Among the fibers, a high amount of fine pores separated from each other can be observed. From the point of view of mechanical properties such a structure is beneficial and a reminder of a construction of some species of plants, e.g., blade of grass, corn stalk. The outer part of the test specimen cross-section transmits and carries external loads that act on the product. The highly porous core behaves as an elastic part, which reduces the shrinkage anisotropy resulting from the melt flow direction. It can also act as a vibration damping element and limit micro-cracks propagation [51,52].

**Figure 11:**
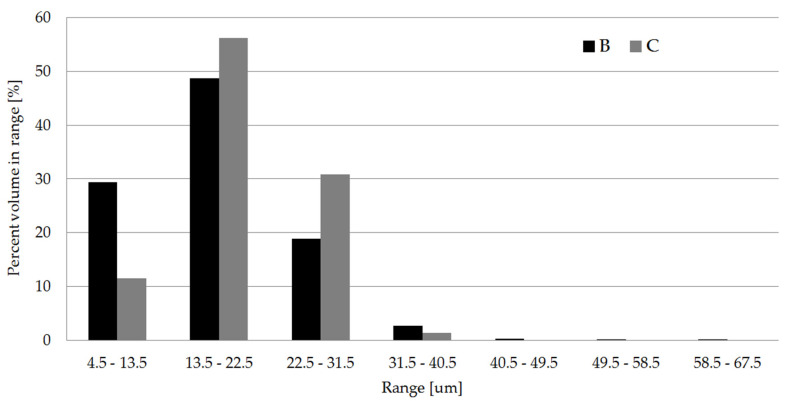
Cell size distribution for test specimens B and C calculated for section II, based on the computed micro tomography analysis.

**Figure 12:**
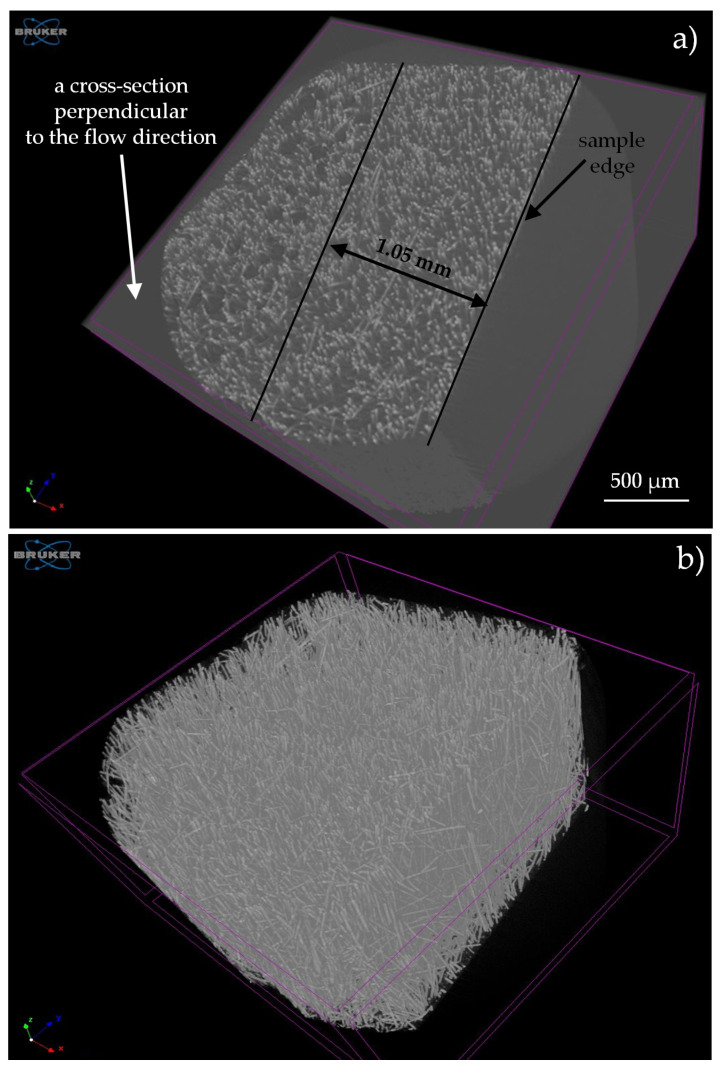
Three-dimensional (3D) view of a fragment of the fibrous-cells structure of sample C in cross-section II: (**a**) A thickness of a non-foamed skin-layer, (**b**) 3D view of fibers oriented in a flow direction.

**Figure 13:**
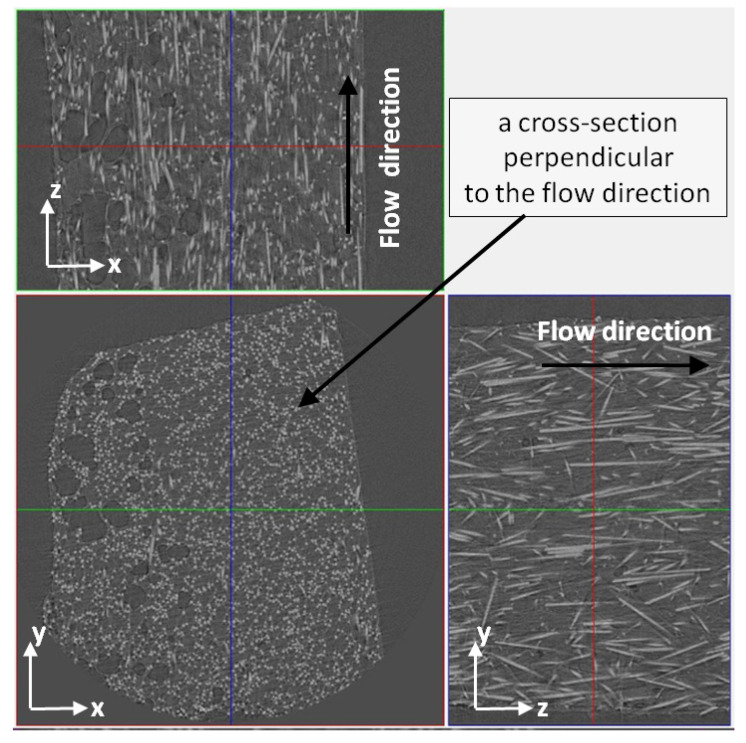
The fibers orientation and effect of cells deformation in the melt flow direction recorded by a high resolution microtomography. The obtained image for sample C in section II.

The observed structural effects were confirmed by computed microtomography images. Based on the tomographic analysis, most of the cells size (99%) is within the range 4.5 and 40.5 µm (see Figure 11). The thickness of the skin-layer for sample C calculated based on SEM images differ insignificantly from the distance between the mold surface, and the first cells in the transition area were registered by micro CT (Figure 12). It can be observed that the short fiber, around 0.4 mm, is oriented in a melt flow direction in a considerable thickness of 3.4 mm (Figure 10 and Figure 13). Actually, this orientation is noticeable in a core area as well, which is surprising taking into account the thickness of the analyzed injection molded part. Registered images allow us to observe that the nitrogen pores are deformed in the flow direction. It is a very valuable supplement to the SEM images. It can be hypothesized that despite the significant sample thickness, the cell growth was limited by the glass fibers arranged in the flow direction of the subsequent layers. The gradient of the fiber arrangement is exactly opposite from the pore distribution. The highest shear stresses occurring in the cavity-melt boundary zone resulted in an even greater orientation of the filler in this zone.

The significant changes in the rheological properties and structure described in the article are reflected in the mass reduction of the molded parts. In all examined cases, the obtained mass reduction of pieces was less than 5.5% in relation to a solid sample A (Table 9). These values were confirmed by CT scans.

## 4. Conclusions

It has been confirmed that the MIM by the MuCell^®^ method significantly affects the apparent viscosity value of the polyamide 66 melt containing 30 wt% of glass fiber.

The lower rheological resistance during the melt flow results from the increase in the shear rate. As a result, the pressure in the mold cavity has been reduced more than four times compared to the solid PA66 GF30 injection. A realization of the filling phase with a reduced filling pressure made it possible to reduce the clamping force, which next leads to a reduction of energy consumed in this phase of the cycle. On the pressure curve in the mold cavity, a second pressure maximum value was recorded, approximately 1.5 s after the end of the injection phase. During this time, the holding pressure was turned off in the injection molding machine. Therefore, it was confirmed that the cells growth process in the molded part takes over the function as the pack and hold pressure in a standard injection molding process. The obtained thick-walled samples B and C were free from warpage or sink marks, although the holding pressure was set to 0.3 s only. Molded pieces with comparable dimensions and a similar weight reduction were obtained.

It was found that in the MuCell^®^ technology, a change in the volume of the mold cavity (the flow cross-section) is a source of additional compression or expansion of the material, as illustrated by changes in pressure in the mold cavity. There was no significant effect of nitrogen pressure (in the tested range) on the location and size of cells, except for the zone close by the gate. Despite the considerable thickness of the samples, regular fine pores of about 20 µm were obtained in the remaining volume of the molded parts.

The gradient in the location of pores was also confirmed, with the thickness of the zone free of them (skin-layer) being 1.4 mm. At the same time, the arrangement of the glass fibers in the direction of flow was confirmed in approximately 80% of the sample volume. Only 20% of the sample volume (layer thickness about 1.6 mm) is the core part with a more disordered arrangement of the fibers.

## Figures and Tables

**Table 1:** Analysis of selected examples of the use of polymers and the thickness of molded parts in the papers concerning the microcellular injection molding (MIM) technology.

Entry in the Literature List	Polymers and Fillers	Molded Part Thickness, mm
[4,7,8,11,13,16,22,23]	PP GF20; PP/LDPE blends; PP + GF + Talc; PP-TPU; PP CF20; PP CF30; PP + EPDM + TALC	2.4–4.0
[6,9,15,17]	PA66 GF 30	1.5–4.0
[7,24]	PLA/PHBV; PLA/PTFE (fibril)	3.3–4.0
[10,12]	PC; PC/ABS	1.7–4.0
[14,25]	PS	3.0

**Table 2:** Process parameters used during parts production in the standard injection molding and MuCell^®^ technology.

Parameters	Standard Injection Molding	MuCell^®^ Technology
filling pressure	58 MPa	70 MPa
switching point *	20 mm	1.0 mm
melt temperature	285 °C	285 °C
holding pressure	28.5 MPa	14 MPa
holding time	7 s	0.3 s
mold temperature	90 °C	90 °C
cooling time	30 s	50 s

* Switching point between the filling-holding phase.

**Table 3:** Gas parameters used during foam parts production.

Type of Sample	Gas Parameters Used in the MuCell^®^ Technology	
	Pressure, MPa	Flow Rate, kg·h^−1^
A *	-	-
B	16	0.36
C	20	0.36

* Injection molding process without gas.

**Table 4:** Shear rate and shear stress changes of the melt in the mold cavity depending on the nitrogen dosing pressure.

**Shear Stress, kPa**		**Type of Sample**		
		**A**	**B**	**C**
ShS1	average value	108.8	82.6	103.4
	standard deviation	22.5	18.4	5.4
ShS2	average value	97.2	110.9	96.5
	standard deviation	6.1	12.8	7.8
**Shear Rate,·s-1**		**Type of Sample**		
		**A**	**B**	**C**
ShR1	average value	270.4	447.4	470.8
	standard deviation	12.0	20.4	14.6
ShR2	average value	252.1	438.6	434.5
	standard deviation	17.8	20.8	30.8

**ShS1**: Shear stress measured at length L1; **ShS2**: Shear stress measured at length L2; **ShR1**: Shear rate measured at length L1; **ShR2**: Shear rate measured at length L2 (Figure 4).

**Table 5:** Apparent viscosity changes of the PA66 GF30 melt measured in the injection mold cavity.

Apparent Viscosity, Pa·s		Type of Sample		
		A	B	C
AV1	average value	221.1	169.6	192.2
	standard deviation	31.2	27,6	19.4
AV2	average value	374.8	246.4	243.0
	standard deviation	39.0	26.0	15.4

AV1: Apparent viscosity measured over distance L1; AV2: Apparent viscosity measured over distance L2 (Figure 4).

**Table 6:** Influence of the MuCell^®^ technology on the maximum pressure of PA66 GF30, measured in the injection mold cavity.

Maximum Pressure in the Mold Cavity, MPa		Type of Sample		
		A	B	C
P_3_	average value	24.3	6.3	7.0
	standard deviation	1.5	0.3	0.5
P_4_	average value	23.9	4.9	6.4
	standard deviation	1.4	0.4	0.6

**Table 7:** Influence of the injection molding method and nitrogen pressure on the maximum temperature of the PA66 GF30 melt, measured in the mold cavity.

Maximum Temperature in the Mold Cavity, °C		Type of Sample Obtained by IM or MuCell^®^ Technology		
		A	B	C
T1	average value	114.2	117.0	118.9
	standard deviation	0.6	1.1	2.6
T2	average value	115.9	112.7	113.1
	standard deviation	2.1	1.4	2.3

**Table 8:** Influence of nitrogen pressure on the average cell size for the foamed PA66 GF30 molded part.

Cell Size, μm		Analyzed Section of Molded Part		
		I	II	III
Sample B	average value	60.5	19.9	19.7
	standard deviation	2.5	4.3	4.5
Sample C	average value	24.8	18.1	19.7
	standard deviation	5.9	3.6	4.7

**Table 9:** Mass reduction of the PA66 GF specimens depending on the nitrogen pressure.

**Density Reduction, %**	**Type of Foamed Sample**		
	Calculated by	**B**	**C**
	Analytical balance	4.5	5.3
	Micro CT	3.3	4.6
